# Phylogenomic approaches to common problems encountered in the analysis of low copy repeats: The sulfotransferase 1A gene family example

**DOI:** 10.1186/1471-2148-5-22

**Published:** 2005-03-07

**Authors:** Michael E Bradley, Steven A Benner

**Affiliations:** 1 Department of Chemistry, University of Florida P.O. Box 117200, Gainesville, FL 32611-7200, USA

## Abstract

**Background:**

Blocks of duplicated genomic DNA sequence longer than 1000 base pairs are known as low copy repeats (LCRs). Identified by their sequence similarity, LCRs are abundant in the human genome, and are interesting because they may represent recent adaptive events, or potential future adaptive opportunities within the human lineage. Sequence analysis tools are needed, however, to decide whether these interpretations are likely, whether a particular set of LCRs represents nearly neutral drift creating junk DNA, or whether the appearance of LCRs reflects assembly error. Here we investigate an LCR family containing the sulfotransferase (SULT) 1A genes involved in drug metabolism, cancer, hormone regulation, and neurotransmitter biology as a first step for defining the problems that those tools must manage.

**Results:**

Sequence analysis here identified a fourth sulfotransferase gene, which may be transcriptionally active, located on human chromosome 16. Four regions of genomic sequence containing the four human SULT1A paralogs defined a new LCR family. The stem hominoid SULT1A progenitor locus was identified by comparative genomics involving complete human and rodent genomes, and a draft chimpanzee genome. SULT1A expansion in hominoid genomes was followed by positive selection acting on specific protein sites. This episode of adaptive evolution appears to be responsible for the dopamine sulfonation function of some SULT enzymes. Each of the conclusions that this bioinformatic analysis generated using data that has uncertain reliability (such as that from the chimpanzee genome sequencing project) has been confirmed experimentally or by a "finished" chromosome 16 assembly, both of which were published after the submission of this manuscript.

**Conclusion:**

SULT1A genes expanded from one to four copies in hominoids during intra-chromosomal LCR duplications, including (apparently) one after the divergence of chimpanzees and humans. Thus, LCRs may provide a means for amplifying genes (and other genetic elements) that are adaptively useful. Being located on and among LCRs, however, could make the human SULT1A genes susceptible to further duplications or deletions resulting in 'genomic diseases' for some individuals. Pharmacogenomic studies of SULT1Asingle nucleotide polymorphisms, therefore, should also consider examining SULT1A copy number variability when searching for genotype-phenotype associations. The latest duplication is, however, only a substantiated hypothesis; an alternative explanation, disfavored by the majority of evidence, is that the duplication is an artifact of incorrect genome assembly.

## Background

Experimental and computational results estimate that 5–10% of the human genome has recently duplicated [1-4]. These estimates represent the total proportion of low-copy repeats (LCRs), which are defined as homologous blocks of sequence from two distinct genomic locations (non-allelic) >1000 base pairs in length. LCRs, which are also referred to in the literature as recent segmental duplications, may contain all of the various sequence elements, such as genes, pseudogenes, and high-copy repeats. A set of homologous LCRs make up an LCR family. Non-allelic homologous recombination between members of an LCR family can cause chromosomal rearrangements with health-related consequences [5-7]. While data are not yet available to understand the mechanistic basis of LCR duplication, mechanisms will emerge through the study of individual cases [8].

At the same time, the appearance of LCR duplicates may be an artifact arising from one of a number of problems in the assembly of a genome of interest. Especially when classical repetitive sequences are involved, it is conceivable that mistaken assembly of sequencing contigs might create in a draft sequence of a genome a repeat where none exists. In the post-genomic world, rules have not yet become accepted in the community to decide when the burden of proof favors one interpretation (a true repeat) over another (an artifact of assembly). Again, these rules will emerge over time through the study of individual cases.

Through the assembly of many case studies, more general features of duplication and evolutionary processes that retain duplicates should emerge. Although each LCR family originates from one progenitor locus, no universal features explain why the particular current progenitor loci have been duplicated instead of other genomic regions. From an evolutionary perspective, duplicated material is central to creating new function, and to speciation. One intriguing hypothesis is that genes whose duplication and recruitment have been useful to meet current Darwinian challenges find themselves in regions of the chromosome that favor the generation of LCRs.

Browsing a naturally organized database of biological sequences, we identified human cytosolic sulfotransferase (SULT) 1A as a recently expanded gene family with biomedically related functions. SULT1A enzymes conjugate sulfuryl groups to hydroxyl or amino groups on exogenous substrates (sulfonation), which typically facilitates elimination of the xenobiotic by the excretory system [9]. Sulfonation, however, also bioactivates certain pro-mutagenic and pro-carcinogenic molecules encountered in the diet and air, making it of interest to cancer epidemiologists [10,11]. These enzymes also function physiologically by sulfonating a range of endogenous molecules, such as steroid and thyroid hormones, neurotransmitters, bile salts, and cholesterol [9].

Three human SULT1A genes have been reported [12,13]. The human SULT1A1 and 1A2 enzymes are ~98% identical and recognize many different phenolic compounds such as *p*-nitrophenol and α-naphthol [14-19]. The human SULT1A3 enzyme is ~93% identical to SULT1A1 and 1A2, but preferentially recognizes dopamine and other catecholamines over other phenolic compounds [19-23]. High resolution crystal structures of SULT1A1 and 1A3 enzymes have been solved [24-26]. Amino acid differences that contribute to the phenolic and dopamine substrate preferences of the SULT1A1 and 1A3 enzymes, respectively, have been localized to the active site [27-30].

Polymorphic alleles of *SULT1A1*, *1A2*, and *1A3 *exist in the human population [31-33]. An allele known as SULT1A1*2 contains a non-synonymous polymorphism, displays only ~15% of wild type sulfonation activity in platelets, and is found in ~30% of individuals in some populations [31]. Numerous studies comparing SULT1A1 genotypes in cancer versus control cohorts demonstrate that the low-activity SULT1A1*2 allele is a cancer risk factor [34-36], although other studies have failed to find an association [12]. Ironically, the protection from carcinogens conferred by the high activity SULT1A1*1 allele is counterbalanced by risks associated with its activation of pro-carcinogens. For example, SULT1A enzymes bioactivate the pro-carcinogen 2-amino-α-carboline found in cooked food, cigarette smoke and diesel exhaust [37]. The sulfate conjugates of aromatic parent compounds convert to reactive electrophiles by losing electron-withdrawing sulfate groups. The resulting electrophilic cations form mutagenic DNA adducts leading to cancer.

Recently, it has become widely understood that placing a complex biomolecular system within an evolutionary model helps generate hypotheses concerning function. This process has been termed "phylogenomics" [38]. Through our bioinformatic and phylogenomic efforts on the sulfotransferase 1A system, we detected a previously unidentified human gene that is very similar to *SULT1A3*, transcriptionally active, and not found in the chimpanzee. In addition, we report that all four human SULT1A genes are located on LCRs in a region of chromosome 16 replete with other LCRs. A model of SULT1A gene family expansion in the hominoid lineage (humans and great apes) is presented, complete with date estimates of three preserved duplication events and identification of the progenitor locus. Positively selected protein sites were identified that might have been central in adapting the SULT1A3 and 1A4 enzymes to their role in sulfonating catecholamines such as dopamine and other structurally related drugs.

## Results and Discussion

### Four human SULT1A genes on chromosome 16 LCRs

**Figure 1:**
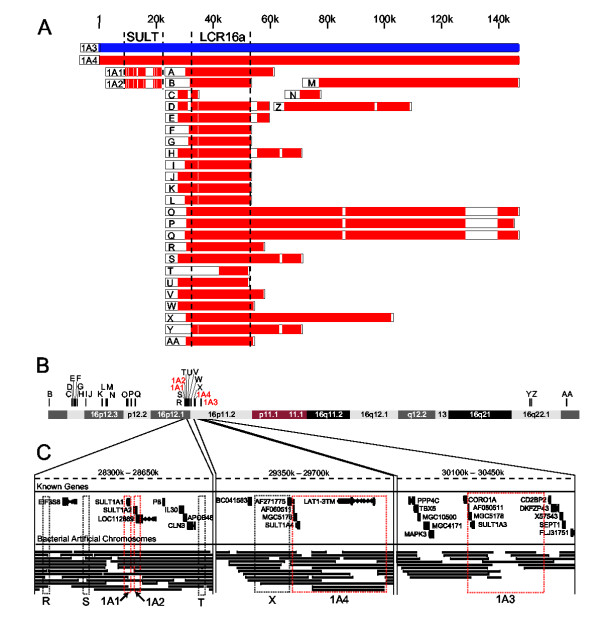
Genomic organization of the SULT1A LCR family. (A) 30 LCRs (red) aligned to the SULT1A3 LCR (blue). Core sequences of SULT1A and LCR16a families are shown between dashed lines. (B) Chromosome 16 positions of 29 SULT1A3-related LCRs. (C) Known genes, bacterial sequencing contigs, and LCRs (outlined in boxes) in three 350 kbp regions of chromosome 16.

The human *SULT1A1 *and *1A2 *genes are tandemly arranged 10 kilobase pairs (kbp) apart in the pericentromeric region of chromosome 16, while the *SULT1A3 *gene is located ~1.7 million base pairs (Mbp) away (Figure 1B and 1C). In addition to the three known SULT1A genes, we found a fourth gene, *SULT1A4*, by searching the human genome with the BLAST-like alignment tool [39]. *SULT1A4 *was located midway between the *SULT1A1*/*1A2 *gene cluster and the *SULT1A3 *gene (Figure 1B and 1C).

The *SULT1A4 *gene resided on 148 kbp of sequence that was highly identical to 148 kbp of sequence surrounding the *SULT1A3 *gene (Figure 1A and Table 1). The high sequence identity between the SULT1A3 and 1A4 genomic regions suggested that they were part of a low copy repeat (LCR) family. This suspicion was confirmed by mining the Recent Segmental Duplication Database of human LCR families [40]. In addition to the four-member SULT1A LCR family, the 148 kbp SULT1A3 LCR was related to 27 other LCRs (Figure 1A and Table 1). Many of the SULT1A3-related LCRs are members of the previously identified LCR16a family [41,42]. The SULT1A3-related LCRs mapping to chromosome 16 collectively amounted to 1.4 Mbp of sequence – or 1.5% of chromosome 16.

**Table 1:** SULT1A3-related LCRs

LCR Name*	Chromosome	Strand	Start	End	Length	% Identity†
A	chr18p	+	11605429	11633851	28422	97.8
B	chr16p	+	11985022	12003971	18949	97.6
C	chr16p	-	14747420	14753000	5580	94.8
D	chr16p	-	14766628	14792117	25489	96.5
E	chr16p	-	14805750	14832437	26687	96.6
F	chr16p	-	14996007	15072649	76642	96.9
G	chr16p	+	15161625	15185467	23842	95.6
H	chr16p	-	15417052	15453865	36813	95.9
I	chr16p	-	16394409	16416404	21995	96.4
J	chr16p	+	16437719	16461029	23310	96.5
K	chr16p	+	18371484	18394809	23325	96.4
L	chr16p	+	18414255	18434928	20673	96.4
M	chr16p	-	18834216	18904410	70194	96.5
N	chr16p	+	18962854	18969729	6875	95.2
O	chr16p	+	21376182	21480283	104101	97.8
P	chr16p	+	21808293	21910109	101816	98.3
Q	chr16p	-	22414809	22523008	108199	97.1
R	chr16p	+	28316465	28341127	24662	97.8
S	chr16p	+	28427424	28467064	39640	97.3
1A1	chr16p	+	28481970	28490644	8674	86.0
1A2	chr16p	+	28494950	28502357	7407	86.6
T	chr16p	-	28621035	28630803	9768	97.7
U	chr16p	-	28692200	28714506	22306	98.1
V	chr16p	-	28800873	28828646	27773	97.7
W	chr16p	-	29084138	29108487	24349	97.8
X	chr16p	+	29426409	29498137	71728	97.6
1A4	chr16p	+	29498152	29644489	146337	99.1
1A3	chr16p	+	30236110	30388351	152241	100
Y	chr16q	+	69784235	69818803	34568	96.2
Z	chr16q	+	70016088	70061019	44931	97.4
AA	chr16q	-	74188141	74209430	21289	97.4

*LCR names are as in Figure 1. †Percent identity is relative to the 1A3 LCR.

To determine if other genes in the SULT super family were also recently duplicated during LCR expansions, we searched the Segmental Duplication Database [4] for human reference genes located on LCRs. No other complete cytosolic SULT genes were located on LCRs, but 25% of the *SULT2A1 *open reading frame (ORF) was located on an LCR (Table 2).

**Table 2:** Duplication Status of SULT Genes

Accession	Gene	Chromosome	ORF Length	ORF Duplicated
NM_001055	*SULT1A1*, phenol	chr16	895	895
NM_001054	*SULT1A2*, phenol	chr16	895	895
NM_003166	*SULT1A3*, dopamine	chr16	895	895
NM_014465	*SULT1B1*	chr4	804	0
NM_001056	*SULT1C1*	chr2	898	0
NM_006588	*SULT1C2*	chr2	916	0
NM_005420	*SULT1E1*	chr4	892	0
NM_003167	*SULT2A1*, DHEA	chr19	864	210
NM_004605	*SULT2B1*	chr19	1059	0
NM_014351	*SULT4A1*	chr22	862	0

The steroid sulfatase gene, which encodes an enzyme that removes sulfate groups from the same biomolecules recognized and sulfonated by SULT enzymes, is frequently deleted in patients with scaly skin (X-linked icthyosis) due to nonallelic homologous recombination between LCRs on chromosome X [43,44]. As demonstrated by the X-linked icthyosis example, SULT1A copy number or activity in the human population could be modified – with health-related consequences – by nonallelic homologous recombination between LCRs on chromosome 16.

### *SULT1A4*: genomic and transcriptional evidence

The sequence of the *SULT1A4 *gene region from the human reference genome was so similar to that of the SULT1A3 region (>99% identity) that the differences were near those that might arise from sequencing error or allelic variation. It was conceivable, therefore, that some combination of sequence error, allelic variation, and/or faulty genome construction generated the appearance of a *SULT1A4 *gene that does not actually exist. We therefore searched for additional evidence that the *SULT1A4 *gene was material.

We asked whether any evidence was consistent with the hypothesis of an artificial SULT1A4 LCR from erroneous genome assembly, as opposed to the existence of a true duplicate region. Here, the quality of the genomic sequencing is important. The junction regions at the ends of the SULT1A4 LCR were sufficiently covered; at least nine sequencing contigs overlapped either junction boundary (Figure 1C). This amount of evidence has been used in other studies to judge the genomic placement of LCRs [45].

As another line of evidence, we compared the nucleotide sequences of the SULT1A4 and 1A3 genomic regions (Table 3). Among the 876 coding positions the only difference was at position 105, where *SULT1A4 *possessed adenine (A) and *SULT1A3 *possessed guanine (G). Thus, if two genes do exist, they differ by one silent transition at the third position of codon 35. The untranslated regions, however, contained thirteen nucleotide differences while the introns contained seven additional differences (Table 3). These 21 differences between the SULT1A4 and 1A3 genomic regions disfavor the hypothesis that sequencing errors played a role in the correct/incorrect placement of these LCRs.

**Table 3:** SULT1A4 and SULT1A3 Genomic Region Differences

Location*	Nucleotide	SULT1A4 Region	SULT1A3 Region
5' UTR	-6,246	G	C
5' UTR	-6,118	C	T
5' UTR	-6,007	G	C
5' UTR	-5,246	-	T
5' UTR	-4,433	-	T
Intron 1B	-2,775	C	T
Intron 1B	-2,671	-	T
Intron 1B	-2,670	-	T
Intron 1B	-2,594	T	G
Intron 1A	-91	-	A
Exon 2	+105	A	G
Intron 4	+853	-	A
Intron 4	+1,487	A	G
Exon 8	+3,569	-	A
Exon 8	+3,570	-	A
Exon 8	+3,571	-	T
Exon 8	+3,572	-	T
3' UTR	+5,379	G	C
3' UTR	+6,438	C	-
3' UTR	+6,335	C	-
3' UTR	+6,210	C	-

* 21 alignment positions are shown where the nucleotide/gapping (-) of the SULT1A4 region differed from that of the SULT1A3 region. Exon and intron names of the *SULT1A3 *gene are according to [33]. All nucleotides are numbered relative to the first nucleotide of the start codon, which has a value of +1. There was no position 'zero'. The last nucleotide of the coding sequence occurs at position +3,188. Approximately 3 kb of upstream (5' UTR) and downstream (3' UTR) nucleotides were included in the comparison.

The *SULT1A4 *gene was located near the junction of two LCRs (Figure 1C). For this reason, it was not clear whether *SULT1A4 *had a functional promoter. We took a bioinformatic approach to address this question. Expressed sequences ascribed to *SULT1A3 *were downloaded from the NCBI UniGene website [46]. Each sequence was aligned to SULT1A3 and SULT1A4 genomic regions. Based on the A/G polymorphism at the third position of codon 35, five expressed sequences were assigned to *SULT1A4 *and nine to *SULT1A3 *(Table 4). Other expressed sequences were unclassified because they did not overlap codon 35. If the *SULT1A4 *does exist, there is ample evidence from expressed sequences to make conclusions about its transcriptional activity.

**Table 4:** Evidence of *SULT1A4 *Expression

Accession	Gene*	Tissue†	Pos. 105
[Genbank:CB147451]	*SULT1A4*	Liver	A
[Genbank:BF087636]	*SULT1A4*	head-neck	A
[Genbank:W76361]	*SULT1A4*	fetal heart	A
[Genbank:W81033]	*SULT1A4*	fetal heart	A
[Genbank:BC014471]	*SULT1A4*	pancreas, epitheliod carcinoma	A
[Genbank:F08276]	*SULT1A3*	infant brain	G
[Genbank:BF814073]	*SULT1A3*	Colon	G
[Genbank:BG819342]	*SULT1A3*	Brain	G
[Genbank:BM702343]	*SULT1A3*	optic nerve	G
[Genbank:BQ436693]	*SULT1A3*	large cell carcinoma	G
[Genbank:AA323148]	*SULT1A3*	cerebellum	G
[Genbank:AA325280]	*SULT1A3*	cerebellum	G
[Genbank:AA349131]	*SULT1A3*	fetal adrenal gland	G
[Genbank:L25275]	*SULT1A3*	placenta	G

*Gene classifications made according to the nucleotide at position 105 as described in the text. ^†^Tissue descriptions were taken from GenBank accessions.

The codon 35 A/G polymorphism was reported as allelic variation in *SULT1A3 *by Thomae *et al*. [33]. It is conceivable that Thomae *et al*. sequenced both *SULT1A3 *and *SULT1A4 *because of the identical sequences surrounding them. In their study, 89% of CAA (1A4) and 11% of CAG (1A3) codon 35 alleles were detected in one population. Why were the frequencies not more equal, as would be expected if *SULT1A4 *is always CAA and *SULT1A3 *is CAG? One hypothesis is that *SULT1A3 *is indeed CAG/CAA polymorphic as reported, while *SULT1A4 *is always CAA. Interestingly, in both the chimpanzee and gorilla, codon 35 of *SULT1A3 *is CAA. This implies that the ancestral *SULT1A3 *gene (prior to duplication) likely had a CAA codon. An A to G transition might have been fixed in a fraction of *SULT1A3 *genes after the divergence of humans and great apes. If this scenario is true, some transcripts assigned to *SULT1A4 *on the basis of codon 35 may actually be from individuals expressing the ancestral CAA version of *SULT1A3*.

### SULT1A progenitor locus

**Figure 2:**
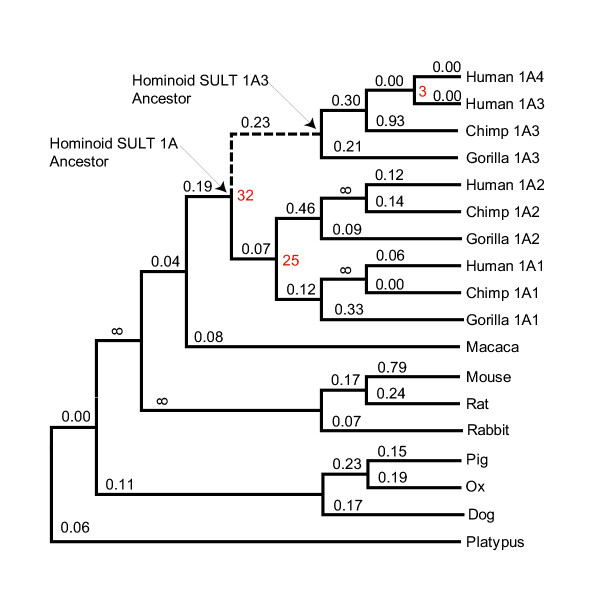
SULT1A gene tree. TREx upper-limit date estimates of hominoid SULT1A duplications are shown as Ma in red. K_A_/K_S _values estimated by PAML are shown above branches. Infinity (8) indicates a non-reliable K_A_/K_S _value greater than 100. The 1A3/1A4 branch is dashed. NCBI accession numbers of sequences used: chimpanzee 1A1 [Genbank:BK004887], chimpanzee 1A2 [Genbank:BK004888], chimpanzee 1A3 [Genbank:BK004889], ox [Genbank:U34753], dog [Genbank:AY069922], gorilla 1A1 [Genbank:BK004890], gorilla 1A2 [Genbank:BK004891], gorilla 1A3 [Genbank:BK004892], human 1A1 [Genbank:L19999], human 1A2 [Genbank:U34804], human 1A3 [Genbank:L25275], human 1A4 [Genbank:BK004132], macaque [Genbank:D85514], mouse [Genbank:L02331], pig [Genbank:AY193893], platypus [Genbank:AY044182], rabbit [Genbank:AF360872], rat [Genbank:X52883].

We aligned the coding sequences of all available SULT1A genes and used various nucleotide distance metrics and tree-building algorithms to infer the gene tree without constraints. The unconstrained topology placed platypus as the out group, with the placental mammals ordered (ox,(pig,(dog,(rodents)),(rabbit,(primates)))). This differed from the topology inferred while constraining for the most likely relationships among mammalian orders (platypus,((dog,(ox,pig)), ((rabbit,(rodents)), primates))) [47]. We considered both trees, and found that the conclusions drawn throughout the paper were robust with regard to these different topologies. Therefore, only the tree inferred while constraining for most likely relationships among mammalian orders is discussed (Figure 2).

Using the transition redundant exchange (TREx) molecular dating tool [48], we placed upper-limit date estimates at the SULT1A duplication nodes (Figure 2). The SULT1A gene family appears to have expanded ~32, 25, and 3 million years ago (Ma). Therefore, the SULT1A duplications likely occurred after the divergence of hominoids and old world monkeys, with the most recent duplication occurring even after the divergence of humans and great apes.

**Figure 3:**
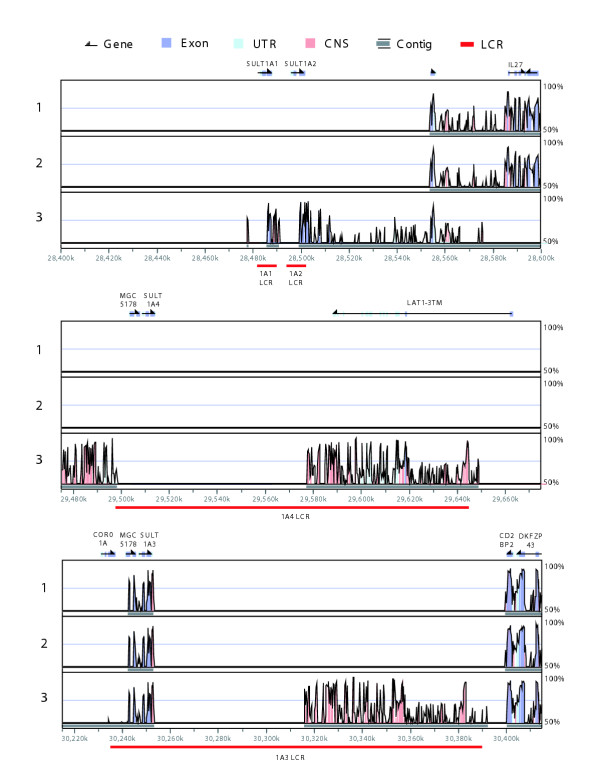
Synteny plots demonstrating *SULT1A3 *is the progenitor locus of the hominoid SULT1A family. Each box shows a VISTA percent identity plot between a section of the human genome and a section of a rodent genome. Different rodent genomes and alignment methods are indicated as follows: 1 = mouse (Oct. 2003 build) multiple alignment method (MLAGAN); 2 = rat (June 2003 build) multiple alignment method (MLAGAN); 3 = mouse (October 2003 build) pairwise alignment method (LAGAN). Human gene locations are shown above and human chromosome 16 coordinates below.

Mouse, rat, and dog genomes each contained a single SULT1A gene. The simplest evolutionary model, therefore, predicted that one of the four hominoid SULT1A loci was orthologous to the rodent *SULT1A1 *gene. Syntenic regions have conserved order of genetic elements along a chromosomal segment and evidence of synteny between homologous regions is useful for establishing relationships of orthology and paralogy. Human *SULT1A1 *is most like rodent *Sult1a1 *in sequence and function and before the advent of whole genome sequencing it was assumed that they were syntenic and therefore orthologous [49]. Complete genome sequences have since emerged and alignments between them are available in the visualization tool for alignments (VISTA) database of human-rodent genome alignments [50,51]. The VISTA database contains mouse-human pairwise alignments and mouse-rat-human multiple alignments. The multiple alignments were found to be more sensitive for predicting true orthologous regions between rodent and human genomes [51]. We searched the VISTA database for evidence of any human-rodent syntenic regions involving the four SULT1A loci. The more sensitive multiple alignments failed to record any human-rodent syntenic regions involving the *SULT1A1*, *SULT1A2*, or *SULT1A4 *loci but detected synteny involving the *SULT1A3 *loci and both rodent genomes (Figure 3). These results are indicative of a hominoid specific SULT1A family expansion from a progenitor locus corresponding to the genomic region that now contains *SULT1A3*. The results from the VISTA database were not as clear when the less sensitive alignment method was employed (Figure 3).

SULT1A3 and 1A4 LCRs were 99.1% identical overall (Table 1). More careful inspection revealed that the SULT1A3 and 1A4 LCRs were 99.8% identical over the first 120 kbp, but only 98.0% identical over the last 28 kbp (data not shown). This 10-fold difference in percent identities (0.2% vs. 2.0%) suggested that the SULT1A4-containing LCR was produced by two independent duplications. The chimpanzee draft genome assembly aligned with the human genome [52] provides evidence in support of this hypothesis. There is conserved synteny between human and chimp genomes over the last 28 kbp of the 1A4-containing LCR, but no synteny over the first 120 kbp where the *SULT1A4 *gene is located (data not shown). This finding and the TREx date estimate for the *SULT1A3*/*1A4 *duplication event at ~3 Ma indicate that *SULT1A4 *is a human invention not shared by chimpanzees – our closest living relatives.

It should be noted that the chimpanzee genome assembly is less reliable than the assembly of the human genome. The coverage is significantly lower, and the methods used for assembly are viewed by many as being less reliable, in part because they relied on the human assembly. Other possibilities, less supported the available evidence, should be considered, including deletion of the chimpanzee *SULT1A4 *gene since the human-chimp divergence, or failure of the draft chimpanzee genome assembly to detect the 120 kbp segment on which the *SULT1A4 *gene resides.

### Adaptive evolution in hominoids

From an analysis of gene sequence change over time, molecular evolutionary theory can generate hypotheses about whether duplication has led to functional redundancy, or whether the duplicates have adopted separate functional roles. If the latter, molecular evolutionary theory can suggest how different the functional roles might be by seeking evidence for positive (adaptive) selection for mutant forms of the native proteins better able to contribute to fitness.

Positive selection of protein function can best be hypothesized when the ratio of non-synonymous (replacement) to synonymous (silent) changes normalized to the number of non-synonymous and synonymous sites throughout the entire gene sequence (K_A_/K_S_) is greater than unity. Various models of evolutionary sequence change can be used to calculate these ratios. The simplest assumes a single K_A_/K_S _ratio over the entire tree (one-ratio). More complex models assume an independent ratio for each lineage (free-ratios), variable ratios for specific classes of sequence sites (site-specific), or variable ratios for specific classes of sequence sites along specified branches (branch-site specific) [53-57].

Estimating the free parameters in each of these models by the maximum likelihood method [58] enables testing two nested evolutionary models as competing hypotheses, where one model is a special case of another model. The likelihood ratio test (LRT) statistic, which is twice the log likelihood difference between the nested models, is comparable to a χ^2 ^distribution with degrees of freedom equal to the difference in free parameters between the models [59]. Evidence for adaptive evolution typically requires a K_A_/K_S _ratio >1 and a statistically significant LRT [60].

We estimated K_A_/K_S _ratios for each branch in the 1A gene tree by maximum likelihood with the PAML program [61]. A typical branch in the SULT1A gene tree had a ratio of 0.16, and the ratio was 0.23 on the branch separating extant *SULT1A3*/*1A4 *genes from the single SULT1A gene in the last common ancestor of hominoids (Figure 2). Thus, the K_A_/K_S _ratio estimated as an average over all sites did not suggest adaptive evolution along the 1A3/1A4 branch.

We then implemented three site-specific and two branch-site evolutionary models that allow K_A_/K_S _ratios to vary among sites. Four of the five models estimated that a proportion of sites (2–8%) had K_A_/K_S _>1 (Table 5). Each model was statistically better at the 99 or 95% confidence level than the appropriate null model as determined using the LRT statistic (Table 6). Table 6 lists the specific sites that various analyses identified as being potentially involved in positive selection and a subset of these sites that are changing along the SULT1A3/1A4 branch.

**Table 5:** Likelihood Values and Parameter Estimates for SULT1A Genes

Model	f.p.*	Log L	Parameter Estimates^†^		
One-ratio	39	- 5,047.81	K_A_/K_S _= 0.15		
Free-ratios	69	- 5,005.18	K_A_/K_S _ratios for each branch shown in Figure 2		
*Site-specific*					
Neutral	36	- 5,021.14	*p*_0 _= 0.48	(*p*_1 _= 0.52)	
			K_A_/K_S 0 _= 0	K_A_/K_S 1 _= 1	
Selection	38	- 4,884.89	*p*_0 _= 0.41	*p*_1 _= 0.13	(*p*_2 _= 0.46)
			K_A_/K_S 0 _= 0	K_A_/K_S 1 _= 1	K_A_/K_S2 _= 0.19
Discrete (k = 2)	37	- 4,931.05	*p*_0 _= 0.68	*p*_1 _= 0.32	
			K_A_/K_S 0 _= 0.06	K_A_/K_S 1 _= 0.77	
Discrete (k = 3)	40	- 4,880.78	*p*_0 _= 0.59	*p*_1 _= 0.33	(*p*_2 _= 0.08)
			K_A_/K_S 0 _= 0.02	K_A_/K_S 1 _= 0.31	**K_A_/K_S2 _= 1.24**
Beta	37	- 4,884.27	*p *= 0.27	*q *= 1.07	
Beta+selection	39	- 4,879.97	*p *= 0.30	*q *= 1.33	
			*p*_0 _= 0.98	*p*_1 _= 0.02	**K_A_/K_S _= > 2.0**
*Branch-site specific*					
Model A	38	- 5,013.29	*p*_0 _= 0.48	*p*_1 _= 0.49	(*p*_2 _= 0.03)
			K_A_/K_S 0 _= 0	K_A_/K_S 1 _= 1	**K_A_/K_S2 _= > 2.0**
Model B	40	- 4,886.52	*p*_0 _= 0.68	*p*_1 _= 0.30	(*p*_2 _= 0.02)
			K_A_/K_S 0 _= 0.04	K_A_/K_S1 _= 0.56	**K_A_/K_S2 _= > 2.0**

*f.p. is the number of free parameters in each model. ^†^Evidence for positive selection is shown in boldface. Proportions of sites in each K_A_/K_S _class, *p*_0_, *p*_1_, and *p*_2_, were not free parameters when in parentheses. Neutral site-specific model assumes two site classes having fixed K_A_/K_S _ratios of 0 and 1, with the proportion of sites in each class estimated as free parameters. Selection site-specific model assumes a third proportion of sites with K_A_/K_S _estimated from the data. Discrete model assumes 2 or 3 site classes (k) with the proportion of sites, and K_A_/K_S _ratios for each proportion, estimated as free parameters. Beta model assumes a beta distribution of sites, where the distribution is shaped by the parameters *p *and *q*. Beta+selection model assumes an additional class of sites having a K_A_/K_S _ratio estimated from the data. Model A, an extension of the neutral model, assumes a third site class on the 1A3/1A4 branch with K_A_/K_S _estimated from the data. Model B, an extension of the discrete model with two site classes (k = 2), also assumes a third site class on the 1A3/1A4 branch with K_A_/K_S _estimated from the data.

**Table 6:** Likelihood Ratio Tests for the SULT1A Genes

	Selection vs. Neutral	Discrete (k = 3) vs. One-ratio	Beta+selection vs. Beta	Model A vs. Neutral	Model B vs. Discrete (k = 2)
Log L_1_	- 4,884.89	- 4,880.78	- 4,879.97	- 5,013.29	- 4,886.52
Log L_0_	- 5,021.14	- 5,047.81	- 4,884.27	- 5,021.14	- 4,931.05
2ΔLog L	272.50	334.06	8.60	15.70	89.06
d.f.	2	4	2	2	2
P-value	P < 0.001	P < 0.001	0.01 < P < 0.05	P < 0.001	P < 0.001
		Positively selected sites*			
		3 (0.86)			
		7 (0.63)			
		30 (0.71)			
		35 (0.73)			
		71 (0.88)			
		77**(0.92)**			
				84**(0.92)**	
		85**(0.95)**			
		86**(0.97)**			
		89**(0.99)**	89 (0.88)	89**(0.99)**	89**(0.99)**
		93**(0.97)**			
				105 (0.72)	105 (0.53)
				107 (0.82)	107 (0.75)
				132 (0.87)	132 (0.78)
				143 (0.51)	
				146 (0.80)	146**(0.97)**
		222**(0.99)**	222 (0.58)		
		236 (0.53)			
		245 **(0.99)**	245 **(0.99)**		
		261 **(0.90)**			
		275 (0.70)			
		288 (0.89)			
		290 **(0.95)**			
		293 (0.72)			

*In parentheses for each positively selected site is the posterior probability that the site belongs to the class with K_A_/K_S _>1. Posterior probabilities >90% are bold-face. Positively selected sites also experiencing non-synonymous change on the 1A3/1A4 branch are underlined.

**Figure 4:**
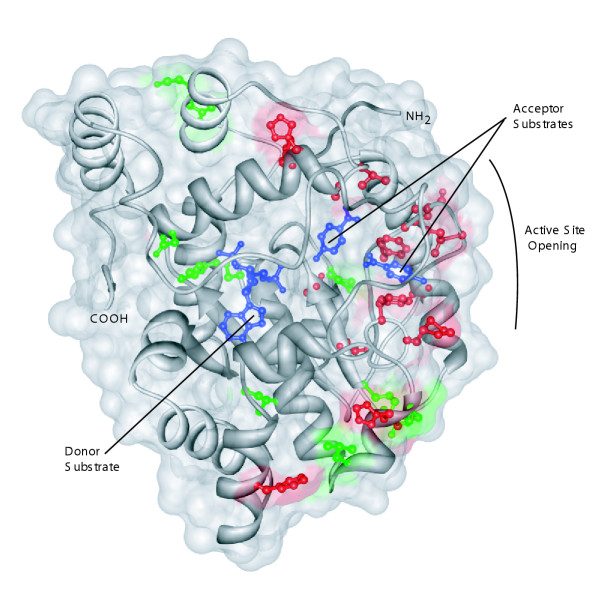
Non-synonymous changes along the 1A3/1A4 branch cluster on the SULT1A1 enzyme structure [PDB: 1LS6] [26]. Red sites experienced non-synonymous changes, green sites experienced synonymous changes. The PAPS donor substrate and *p*-nitrophenol acceptor substrates are shown in blue. Image was generated using Chimera [86].

A hypothesis of adaptive change that is based on the use of K_A_/K_S_values can be strengthened by joining the molecular evolutionary analysis to an analysis based on structural biology [62,63]. Here, we ask whether the sites possibly involved in an episode of sequence evolution are, or are not, randomly distributed in the three dimensional structure. To ask this question, we mapped the sites to the SULT1A structure (Figure 4). Sites holding amino acids whose codons had suffered synonymous replacements were evenly distributed throughout the three-dimensional structure of the enzyme, as expected for silent changes that have no impact on the protein structure and therefore cannot be selected for or against at the protein level (Figure 4). In contrast, sites experiencing non-synonymous replacements during the episode following the duplication that created the new hominoid gene are clustered on the side of the protein near the substrate binding site and the channel through which the substrate gains access to the active site (Figure 4 and Table 7). This strengthens the hypothesis that replacements at the sites are indeed adaptive. The approach employed here based on structural biology does not lend itself easily to evaluation using statistical metrics. Rather, the results are valuable based on the visual impression that they give, and the hypotheses that they generate.

**Table 7:** Non-synonymous Changes on the 1A3/1A4 Branch

Site*	Nucleotide Changes/Site	Hominoid SULT1A Ancestor				Hominoid SULT1A3 Ancestor		
				
		Residue	PP^†^	Physicochemical Properties		Residue	PP	Physicochemical Properties
44	1	Ser	(1.00)	tiny polar	→	Asn	(1.00)	small polar
71	1	His	(0.99)	non-polar aromatic positive	→	Asn	(1.00)	small polar
76	1	Phe	(1.00)	non-polar aromatic	→	Tyr	(1.00)	aromatic
77	2	Met	(0.99)	non-polar	→	Val	(1.00)	small non-polar aliphatic
84	1	Phe	(1.00)	non-polar aromatic	→	Val	(1.00)	small non-polar aliphatic
85	1	Lys	(1.00)	Positive	→	Asn	(1.00)	small polar
86	2	Val	(0.98)	small non-polar aliphatic	→	Asp	(1.00)	small polar negative
89	3	Ile	(0.98)	non-polar aliphatic	→	Glu	(1.00)	polar negative
93	1	Met	(0.00)	non-polar	→	Leu	(1.00)	non-polar aliphatic
101	1	Ala	(1.00)	tiny non-polar	→	Pro	(1.00)	small
105	1	Leu	(1.00)	non-polar aliphatic	→	Ile	(1.00)	son-polar aliphatic
107	1	Thr	(1.00)	tiny polar	→	Ser	(1.00)	tiny polar
132	1	Ala	(1.00)	tiny non-polar	→	Pro	(1.00)	small
143	1	Tyr	(1.00)	aromatic	→	His	(1.00)	non-polar aromatic positive
144	2	His	(0.99)	non-polar aromatic positive	→	Arg	(1.00)	polar positive
146	2	Ala	(1.00)	tiny non-polar	→	Glu	(1.00)	polar negative
148	1	Val	(1.00)	small non-polar aliphatic	→	Ala	(1.00)	tiny non-polar
222	1	Leu	(0.99)	non-polar aliphatic	→	Phe	(1.00)	non-polar aromatic

* Sites underlined were identified as being positively selected using the branch-site specific models. ^†^Posterior probabilities that the ancestral residues are correct, conditional on the model of sequence evolution used.

We then examined literature where amino acids had been exchanged between SULT1A1 and SULT1A3. One of the sites, at position 146, identified as being involved in adaptive change, is known to control substrate specificity in SULT1A1 and 1A3 [27-30]. The remaining sites identified are nearby.

## Conclusion

An interesting question in post-genomic science asks how to create biological hypotheses from various drafts of whole genome sequences. In generating these hypotheses, it is important to remember that a genomic sequence is itself a hypothesis, about the chemical structure of a small number of DNA molecules. In many cases, biologists wish to move from the genomic sequence, as a hypothesis, to create hypotheses about biological function, without first "proving" the genome sequence hypothesis.

This type of process, building hypotheses upon unproven hypotheses, is actually common in science. In fact, very little of what we believe as fact is actually "proven"; formal proof is virtually unknown in science that involves observation, theory, and experiment. Rather, scientists generally accumulate data until a burden of proof is met, with the standards for that burden being determined by experience within a culture. In general, scientists have an idea in an area as to what level of validation is sufficient to avoid making mistakes an unacceptable fraction of the time, and proceed to that level in their ongoing work, until they encounter a situation where they make a mistake (indicating that a higher standard is needed), or encounter enough examples where a lower standard works, and therefore come to accept a lower standard routinely [64].

Genomics has not yet accumulated enough examples for the culture to define the standards for a burden of proof. In the example discussed here, several lines of reasoning would be applied to analyze the sulfotransferase gene family. First, the fact that the draft genome for chimpanzee contains three paralogs, while the draft genome for human contains four, would normally be interpreted (as it is here) as evidence that an additional duplication occurred in the time since chimpanzee and humans diverged. It would also, however, be consistent with the loss of one of four hypothetical genes present in the common ancestor of chimpanzee and humans in the lineage leading to chimpanzee. Another possibility is that the finishing stages of the chimpanzee genome project will uncover a *SULT1A4 *gene.

Normally, one would resolve this question using an out group taxon, a species that diverged from the lineage leading to chimpanzee and human before chimpanzee and human themselves diverged. The nearest taxa that might serve as an out group today are, however, rat and mouse. As noted above, they diverged so long ago (*ca*. 150 MY separates contemporary rodents from contemporary primates) that the comparison provides no information. And no closer out group taxon (*e.g*., orangutan) has had its genome completely sequenced.

Here, the two hypotheses (duplication versus loss after the chimpanzee-human speciation) are distinguished (to favor post-speciation duplication) based on an analysis of the silent nucleotide substitutions using the TREx metric. The very small number of nucleotide differences separating the *SULT1A3 *and *SULT1A4 *coding regions favors the generation of the two paralogs after chimpanzee and human diverged.

This comparison, however, potentially suffers from the statistics of small numbers. The number of differences in the coding region (exactly one) is small. By considering ~10 kbp of non-coding sequence, however, additional differences were found. It is possible that in the assembly of the human genome, a mistake was made that led to the generation of a SULT1A4 region that does not actually exist. In this hypothesis, the ~20 nucleotide differences between the *SULT1A3 *and *SULT1A4 *paralogs must be the consequence of allelic polymorphism in the only gene that exists. This is indeed how some of the data were initially interpreted.

Does the preponderance of evidence favor the hypothesis of a very recent duplication to generate a pair of paralogs (*SULT1A3 *and *SULT1A4*)? Or does the evidence favor the hypothesis that the *SULT1A4 *gene is an illusion arising from gene assembly error coupled to sequencing errors and/or allelic variation at ca. 20 sites? The culture does not yet have a standard of assigning the burden of proof here, although a choice of hypothesis based simply on the count of the number of mistakes that would need to have been made to generate each hypothesis (none for the first, at least three for the second) would favor the former over the latter. Thus, perhaps naively, the burden of proof now favors the former, and we may proceed to generate the biological hypothesis on top of the genomic hypothesis.

Here, the hypothesis has immediate pharmacogenomic and genomic disease implications due to the specific functional behaviors of SULT1A enzymes. LCR-mediated genomic rearrangements could disrupt or amplify human SULT1A gene copy number. Given our current environmental exposure to many forms of carcinogens and pro-carcinogens that are either eliminated or activated by SULT enzymes, respectively, it is plain to see how SULT1A copy number variability in the human population could underlie cancer susceptibilities and drug or food allergies.

The majority of evidence indicates that a new transcriptionally active human gene, which we refer to as *SULT1A4*, was created when 120 kbp of chromosome 16 duplicated after humans diverged from great apes. Thus, *SULT1A4*, or possibly another gene in this region, is likely to contribute to distinguishing humans from their closest living relatives. It is also conceivable that an advantage in gene regulation, as opposed to an advantage from gene duplication, was the driving force behind the duplication of this 120 kbp segment. While cause and effect are difficult to separate, the examples presented here support the hypothesis that genes whose duplication and recruitment are useful to meet current Darwinian challenges find themselves located on LCRs.

The *SULT1A4 *gene is currently the most obvious feature of the duplicated region and has been preserved for ~3 MY without significant divergence of its coding sequence. One suggestion for the usefulness of *SULT1A4 *is that it expanded sulfonating enzymes to new tissues. The *SULT1A4 *gene is located only 10 kbp upstream from the junction boundary of its LCR and 700 kbp away from the *SULT1A3 *locus. It is possible, therefore, that promoter elements from the new genomic context of the *SULT1A4 *gene would drive its expression in tissues where *SULT1A3 *is not expressed – a hypothesis testable by more careful transcriptional profiling.

Multiple SULT1A genes were apparently useful inventions by our stem hominoid ancestor. Following the duplication of an ancestral primate SULT1A gene ~32 Ma, positive selection acted on a small proportion of sites in one of the duplicates to create the dopamine sulfonating SULT1A3 enzyme. In the example presented here, the evidence of adaptive change at certain sites is corroborated by the *ad hoc *observation that the sites cluster near the active site of the protein. The well known substrate binding differences at the active sites of SULT1A1/1A2 and SULT1A3 (and now SULT1A4) substantiate these findings.

When studying well-characterized proteins as we have done here, episodes of functional change can be identified by piecing together several lines of evidence. It is not immediately possible, unfortunately, to assemble as much evidence for the majority of proteins in the biosphere. Thus, an important goal in bioinformatics is to recognize the signal of functional change from a restricted amount of evidence. Of the three lines of evidence employed here (codon-based metrics, structural biology, and experimental), structural biology, with its obvious connections to protein function and impending growth from structural genomics initiatives, will probably be the most serviceable source of information for most protein families. This should be especially true for protein families not amenable to experimental manipulation, or with deep evolutionary branches where codon-based metrics are unhelpful. If we are to exploit the incontrovertible link between structure and function, however, new structural bioinformatic tools and databases relating protein structure to sequence changes occurring on individual branches are much needed.

This bioinformatic study makes several clear predictions. First, a PCR experiment targeted against the variation between the hypothetical *SULT1A3 *and *SULT1A4 *human genes should establish the existence of the two separate genes. Second, a reverse transcription-PCR experiment would be expected to uncover transcriptional activity for the *SULT1A3 *and *SULT1A4 *human genes. Since this paper was submitted, these experiments have been done, and indeed confirm our predictions made without the experimental information [65]. Further, after this manuscript and its computationally-based predictions were submitted for publication, a largely finished sequence for chromosome 16 has emerged [66] that confirms our analysis here in every respect.

## Methods

### SULT1A LCR family organization in the human genome

The July 2003 human reference genome (based on NCBI build 34) was queried with the *SULT1A3 *coding region using the BLAST-like alignment tool [39], and search results were visualized in the UCSC genome browser [67]. Two distinct locations on chromosome 16 were identified as equally probable. One location was recognized by NCBI Map Viewer [68] as the *SULT1A3 *locus. The other locus was dubbed *SULT1A4 *following conventional naming for this family. The coding sequence and genomic location of *SULT1A4*, as well as expressed sequences derived from *SULT1A4*, have been deposited with the GenBank Third Party Annotation database under accession [Genbank:BK004132].

To determine the extent of homology between the SULT1A3 and 1A4 genomic locations, ~500 kbp of sequence surrounding *SULT1A3 *and ~500 kbp of sequence surrounding *SULT1A4 *were downloaded from NCBI and compared using PIPMAKER [69,70]. Before submitting to PIPMAKER, high-copy repeats in one of the sequences were masked with REPEATMASKER [71].

The Human Recent Segmental Duplication Page [72] was consulted to identify other LCRs related to the SULT1A3-containing LCR. Chromosomal coordinates of 30 SULT1A3-related LCRs were arranged in GFF format and submitted to the UCSC genome browser as a custom track. Sequences corresponding to the chromosomal coordinates of the 30 LCRs were then downloaded from the UCSC genome browser and parsed into separate files. Each LCR was aligned with the SULT1A3-containing LCR using MULTIPIPMAKER [73]. The Segmental Duplication Database [74] was used to examine the duplication status of each gene in the cytosolic SULT super family.

The bacterial artificial chromosome contigs supporting each member of the SULT1A LCR family, and the known genes within each LCR, were inspected with the UCSC genome browser [75]. The DNA sequences of nine bacterial artificial chromosome contigs supporting the SULT1A4 genomic region [NCBI Clone Registry: CTC-446K24, CTC-529P19, CTC-576G12, CTD-2253D5, CTD-2324H19, CTD-2383K24, CTD-2523J12, CTD-3191G16, RP11-28A6] and seven contigs supporting the SULT1A3 region [NCBI Clone Registry: CTD-2548B1, RP11-69O13, RP11-164O24, RP11-455F5, RP11-612G2, RP11-787F23, RP11-828J20] were downloaded from the UCSC genome browser website.

### Phylogenetics

The MASTERCATALOG was used for performing initial inspections of the SULT gene family and for delivering a non-redundant collection of SULT1A genes. Additional SULT1A ORFs were extracted from gorilla working draft contigs [Genbank:AC145177] (*SULT1A1 *and *1A2*) and [Genbank:AC145040] (*SULT1A3*) and chimpanzee whole genome shotgun sequences [Genbank:AACZ01082721] (*SULT1A1*), [Genbank:AADA01101065] (*SULT1A2*), and [Genbank:AACZ01241716] (*SULT1A3*) using PIPMAKER exon analysis. These new SULT1A genes have been deposited with the GenBank Third Party Annotation database under accession numbers [Genbank:BK004887-BK004892]. DNA sequences were aligned with CLUSTAL W [76]. The multiple sequence alignment used in all phylogenetic analyses is presented as supplementary data [see Additional file 1]. Pairwise distances were estimated under various distance metrics (Jukes-Cantor, Kimura 2-parameter, and Tamura-Nei) that account for among-site rate variation using the gamma distribution [77]. Phylogenies were inferred using both neighbor-joining and minimum evolution tree-building algorithms under the following constraints ((((primates), rodents), (artiodactyls, carnivores)), platypus). Phylogenetic analyses were conducted using the MEGA2 v2.1 [78] and PAUP* v4.0 [79] software packages.

Parameter estimates of site class proportions, K_A_/K_S _ratios, base frequencies, codon frequencies, branch lengths, and the transition/transversion bias were determined by the maximum likelihood method with the PAML v3.14 program [61]. Positively selected sites, posterior probabilities, and marginal reconstructions of ancestral sequences were also determined using PAML. Sites experiencing synonymous changes along the 1A3/1A4 branch were recorded by hand from an ancestral sequence alignment.

### Molecular dating

Starting with aligned DNA sequences, the number (*n*) of two-fold redundant codons (Lys, Glu, Gln, Cys, Asp, Phe, His, Asn, Tyr) where the amino acid had been conserved in pairs of aligned sequences, and the number of these codons where the third position was identically conserved (*c*) were counted by the DARWIN bioinformatics platform [80,81]. The pairwise matrix of *n *and *c *values for all SULT1A genes is presented as supplementary data [see Additional file 2]. The *c*/*n *quotient equals the fraction of identities (*f*_2_) in this system, or the transition redundant exchange (TREx) value [48]. TREx values were converted to TREx distances (*kt *values) by the following equation: *kt *= - ln [(*f*_2 _- *Eq*.) / (1 - *Eq*.)], where *k *is the rate constant of nucleotide substitution, *t *is the time separating the two sequences, and *Eq*. is the equilibrium state of the TREx value [48]. The equilibrium state of the TREx value was estimated as 0.54 for primates, and the rate constant at two-fold redundant sites where the amino acid was conserved (*k*) was estimated as 3.0 × 10 ^-9 ^changes/site/year for placental mammals (T. Li, D. Caraco, E. Gaucher, D. Liberles, M. Thomson, and S.A.B., unpublished data). These estimates were determined by sampling all pairs of mouse:rat and mouse:human orthologs in the public databases and following accepted placental mammal phylogenies and divergence times [82,83]. Therefore, the date estimates reported are based on the contentious assumptions that (i) rates are constant at the third position of two-fold redundant codons across the genome, (ii) the fossil calibration points are correct, and (iii) the mammalian phylogeny used is correct. Branch lengths were obtained for the constrained tree topology from the pairwise matrix of TREx distances using PAUP* v4.0. Upper-limit date estimates for nodes corresponding to SULT1A duplication events were obtained by summing the longest path of branches leading to a node and dividing that value by *k*.

### Comparative genomics

Human-chimpanzee genome alignments were inspected at the UCSC Genome Browser. Human-rodent genome alignments were examined with the VISTA Genome Browser [50,51,84]. VISTA default parameters were used for drawing curves. Alignments constructed using both the pairwise method (LAGAN) and the multiple alignment method (MLAGAN) between the human genome builds frozen on April 2003 or July 2003 and both rodent genomes were inspected.

### Transcriptional profiling

All expressed sequences ascribed to *SULT1A3 *were downloaded from NCBI UniGene [85] and aligned with SULT1A3 and SULT1A4 genomic regions using PIPMAKER. Alignments were inspected for the polymorphism in codon 35, as well as any other potential patterns, to determine whether they were derived from *SULT1A3 *or *SULT1A4*.

## Abbreviations

kbp (kilobase pairs); LCR (low copy repeat); Mbp (million base pairs); Ma (million years ago); ORF (open reading frame); SULT (sulfotransferase); TREx (transition redundant exchange); VISTA (visualization tool for alignments).

## Authors' contributions

M.E.B carried out the study and drafted the manuscript. S.A.B participated in designing the study and preparing the manuscript.

## Supplementary Material

Additional File 1 Multiple sequence alignment of SULT1A genes.Multiple sequence alignment of SULT1A genes used in all phylogenetic analyses. Characters conserved in all sequences are indicated with asterisks. Click here for file

Additional File 2 Pairwise *n *and *c *values for SULT1A genes. Pairwise *n *and *c *values between SULT1A genes. The names of the sequences are the row-headers and the column-headers. Lower triangular matrix contains *n *values, and upper triangular matrix contains *c *values. Click here for file
