# Supplementary material for: Phylogenomic approaches to common problems encountered in the analysis of low copy repeats: The sulfotransferase 1A gene family example
Source: BMC Evol Biol. 2005 Mar 7;5:22. doi: 10.1186/1471-2148-5-22 (PMC555591; doi:10.1186/1471-2148-5-22)
Supplement: Additional File 1 — Multiple sequence alignment of SULT1A genes.Multiple sequence alignment of SULT1A genes used in all phylogenetic analyses. Characters conserved in all sequences are indicated with asterisks. [file 1471-2148-5-22-S1.pdf]

```
            ****                          *   *    **          ** ** **  * ** ** ***** *****
   Human1A4 ATGGAG---------CTGATCCAGGACACCTCCCGCCCGCCACTGGAGTACGTGAAGGGGGTCCCGCTCATCAAGTACTT    71
   Human1A3 ATGGAG---------CTGATCCAGGACACCTCCCGCCCGCCACTGGAGTACGTGAAGGGGGTCCCGCTCATCAAGTACTT    71
   Chimp1A3 ATGGAG---------CTGATCCAGGACACCTCCCGCCCGCCACTGGAGTACGTGAAGGGGGTCCCGCTCATCAAGTACTT    71
 Gorilla1A3 ATGGAG---------CTGATCCAGGACACCTCCTGCCCACCACTGGAGTACGTGAAGGGGGTCCCGCTCATCAAGTACTT    71
   Human1A2 ATGGAG---------CTGATCCAGGACATCTCTCGCCCGCCACTGGAGTACGTGAAGGGGGTCCCGCTCATCAAGTACTT    71
   Chimp1A2 ATGGAG---------CTGATCCAGGACACCTCCCGCCCGCCACTGGAGTACGTGAAGGGGGTCCCGCTCATCAAGTACTT    71
 Gorilla1A2 ATGGAG---------CTGATCCAGGACATCTCTCGCCCGCCACTGGAGTACGTGAAGGGGGTCCCGCTCATCAAGTACTT    71
   Human1A1 ATGGAG---------CTGATCCAGGACACCTCCCGCCCGCCACTGGAGTACGTGAAGGGGGTCCCGCTCATCAAGTACTT    71
   Chimp1A1 ATGGAG---------CTGATCCAGGACACCTCCCGCCCGCCACTGGAGTACGTGAAGGGGGTCCCGCTCATCAAGTACTT    71
 Gorilla1A1 ATGGAG---------CTGATCCAGGATATCTCCCGCCTGCCACTGGAGTACGTGAAGGGGGTCCCGCTCATCAAGTACTT    71
  Macaca1A1 ATGGAG---------CTGATCCAGGACACCTCTCGCCCGCCACTGGAGTATGTGAAGGGGGTTCCGCTCATCAAGTACTT    71
  Rabbit1A1 ATGGAG---------CTCATCCAGGACACGTCCCGCCCACCGCTGGAGTACGTGAAGGGGGTCCCGCTCATCAAGTACTT    71
     Dog1A1 ATGGAG---------GACATTCCCGACACCTCGCGCCCACCACTGAAGTATGTGAAGGGGATCCCTCTAATCAAGTACTT    71
   Mouse1A1 ATGGCTCAGAACCCCAGCAACATGGAGCCCTTGCGTAAACCACTGGTGCCTGTGAAGGGAATCCCACTCATCAAATACTT    80
     Rat1A1 ATGGAG---------------------TTCTCCCGTCCACCGCTAGTGCATGTGAAGGGTATCCCACTCATCAAATACTT    59
     Cow1A1 ATGGAA---------CTGATCCAGGATACCTCCCGCCCTCCAGCAAAGTATGTGAAGGGCATCCCTCTCATCAAGTACTT    71
     Pig1A1 ATGGAG---------CCGGTCCAGGACACCTACCGCCCGCCACTGGAGTACGTGAAGGGGGTCCCTCTCATCAAGTACTT    71
Platypus1A1 ATGGAA---------CTGTCTCCGGACACGTCGCGCCCACCCGTGGTACGGGTCAACGGCATCCCTCTCATCAAATACTT    71
      ruler 1.......10........20........30........40........50........60........70........80

             ** ***    * *      ** *    **    *     **   ***  * **  **  *** ** ** ** ** ** *
   Human1A4 TGCAGAGGCACTGGGGCCCCTGCAGAGCTTCCAAGCCCGACCTGATGACCTGCTCATCAACACCTACCCCAAGTCTGGCA   151
   Human1A3 TGCAGAGGCACTGGGGCCCCTGCAGAGCTTCCAGGCCCGACCTGATGACCTGCTCATCAACACCTACCCCAAGTCTGGCA   151
   Chimp1A3 TGCAGAGGCACTGGGGCCCCTGCAGAGCTTCCAAGCCCGACCTGATGACCTGCTCATCAACACCTACCCCAAGTCTGGCA   151
 Gorilla1A3 TGCAGAGGCACTGGGGCCCCTGCAGAGCTTCCAAGCCCGACCTGATGACCTGCTCATCAACACCTACCCCAAGTCTGGCA   151
   Human1A2 TGCAGAGGCACTGGGGCCCCTGCAGAGCTTCCAGGCCCGGCCTGATGACCTGCTCATCAGCACCTACCCCAAGTCCGGCA   151
   Chimp1A2 TGCAGAGGCACTGGGGCCCCTGCAAAGCTTCCAGGCCCGGCCTGATGACCTGCTCATCAGCACCTACCCCAAGTCCGGCA   151
 Gorilla1A2 TGCAGAGGCACTGGGGCCCCTGCAGAGCTTCCAGGCCTGGCCCGATGACCTGCTCATCAGCACCTACCCCAAGTCCGGCA   151
   Human1A1 TGCAGAGGCACTGGGGCCCCTGCAGAGCTTCCAGGCCCGGCCTGATGACCTGCTCATCAGCACCTACCCCAAGTCCGGCA   151
   Chimp1A1 TGCAGAGGCACTGGGGCCCCTGCAAAGCTTCCAGGCCCGGCCTGATGACCTGCTCATCAGCACCTACCCCAAGTCCGGCA   151
 Gorilla1A1 TGCAGAGGCACTGGGGTCCCTGCAGAGCTTCCAGGCCCGGCCTGATGACCTGCTCATCAGCACCTACCCCAAGTCCGGCA   151
  Macaca1A1 TGCAGAGGCACTGGGACCCCTGCAGAGCTTCCGGGCCCGGCCCGATGACCTGCTCATCAGCACCTACCCCAAGTCCGGGA   151
  Rabbit1A1 TGCTGAGGCGATGGGGCCACTGCAAAGCTTCAAGGCCCGGCCCGATGACCTGCTCATCAGCACCTACCCCAAGTCCGGCA   151
     Dog1A1 TGCAGAGGCACTGGAGTCACTGCAGGACTTCCAAGCTCAGCCTGATGACCTGCTTATCAGCACGTACCCCAAATCTGGCA   151
   Mouse1A1 TGCGGAGACAATGGAGCAACTGCAGAACTTCACAGCCTGGCCTGATGATGTGCTCATCAGCACGTACCCAAAGTCTGGTA   160
     Rat1A1 TGCAGAGACAATTGGGCCATTGCAGAACTTCACAGCCTGGCCTGATGACTTGCTGATCAGCACATACCCAAAGTCTGGTA   139
     Cow1A1 TGCAGAGGCCCTGGGGCCACTGGAGAGCTTTGAAGCCTGGCCTGATGACTTGCTCATCAGCACCTACCCCAAGTCGGGCA   151
     Pig1A1 TGCAGAGGCACTGGGGCCACTGGAGAGTTTCCAAGCTTGGCCCGATGACGTGCTGATCAGCACCTATCCCAAATCTGGTA   151
Platypus1A1 CGCTGAGAACTTGGGGGCACTGGAGAGCTTGCGGGTCCAGCCCAGTGACCTCCTTGTCTCCACCTACCCCAAATCCGGCA   151
      ruler ........90.......100.......110.......120.......130.......140.......150.......160

            * *  *** * **  * **  * **  * ***** ** *  **  *     * *****     * *  **  ***
   Human1A4 CCACCTGGGTGAGCCAGATACTGGACATGATCTACCAGGGCGGCGACCTAGAGAAGTGTAACCGGGCTCCCATCTACGTA   231
   Human1A3 CCACCTGGGTGAGCCAGATACTGGACATGATCTACCAGGGCGGCGACCTAGAGAAGTGTAACCGGGCTCCCATCTACGTA   231
   Chimp1A3 CCACCTGGGTGAGCCAGATACTGGACATGATCTACCAGGGCGGCGACCTACAAAAGTGTAACCGGGTTCCCATCTACGTA   231
 Gorilla1A3 CCACCTGGGTGAGCCAGATACTGGACATGATCTACCAGGGCGGCAACCTAGAGAAGTGTAACCGGGCTCCCATCTACGTA   231
   Human1A2 CCACCTGGGTGAGCCAGATTCTGGACATGATCTACCAGGGCGGTGACCTGGAAAAGTGTCACCGAGCTCCCATCTTCATG   231
   Chimp1A2 CCACCTGGGTGAGCCAGATTTTGGACATGATCTACCAGGGTGGTGACCTGGAGAAGTGTCACCGAGCTCCCATCTACATG   231
 Gorilla1A2 CCACCTGGGTAAGCCAGATTCTGGACATGATCTACCAGGGCGGTGACCTGGAGAAGTGTCACCGAGCTCCCATCTTCATG   231
   Human1A1 CTACCTGGGTAAGCCAGATTCTGGACATGATCTACCAGGGTGGTGACCTGGAGAAGTGTCACCGAGCTCCCATCTTCATG   231
   Chimp1A1 CCACCTGGGTGAGCCAGATTCTGGACATGATCTACCAGGGCGGTGACCTGGAGAAGTGTCACCGAGCTCCCATCTTCATG   231
 Gorilla1A1 CCACCTGGGTGAGCCAGATTGTGGACATGATCTACCAGGGCGGCGATCTGGAGAAGTGTCACCGAGCTCCCATCTTCATG   231
  Macaca1A1 CTACCTGGGTGAGCCAGATTCTGGACATGATCTACCAAGGTGGCGACCTGGAGAAGTGTCGCCGAGCTCCCATCTTCATG   231
  Rabbit1A1 CTACCTGGGTGAGCGAGATACTGGATATGATCTACCAGGCCGGTGACCAACAGAAGTGTCTCCGGGCCCCCATCTACATC   231
     Dog1A1 CCACTTGGGTGAGCGAGATCCTGGACATGATCTACCAAGACGGTGATGTAGAGAAGTGTCGCAGGGCCCCCGTCTTCATC   231
   Mouse1A1 CTAACTGGATGAGTGAGATCATGGATATGATCTATCAGGGTGGCAAGCTAGATAAGTGTGGCCGGGCCCCCGTCTATGCC   240
     Rat1A1 CTACCTGGATGAGTGAGATCCTGGATATGATCTATCAGGGTGGCAAGCTAGAGAAGTGTGGCCGCGCCCCCATCTATGCC   219
     Cow1A1 CCACCTGGGTAAGCGAGATCCTGGACCTGATCTACCAGGAGGGTGACCTGGAGAAGTGTCAGAGAGCCCCTGTCTTCTTG   231
     Pig1A1 CCACCTGGGTGAGCGAGATCCTGGACCTAATCTACCAGGGTGGTGACCTGCAGAAGTGTCAGAGAGCCCCCATCTTTGTG   231
Platypus1A1 CCACGTGGGTGAGCGAAATCCTCGACATGATCTACCAGGGAGGAGACCTAGAGAAGTGCCAACGAGCGCCTGTCTTCTTA   231
      ruler .......170.......180.......190.......200.......210.......220.......230.......240
```

```
             *** * ** ** ** *** ***     **  *    *** ****  * **     *  *  * **  *  * ** **  *
   Human1A4 CGGGTGCCCTTCCTTGAGGTCAATGATCCAGGGGAACCCTCAGGGCTGGAGACTCTGAAAGACACACCGCCCCCACGGCT   311
   Human1A3 CGGGTGCCCTTCCTTGAGGTCAATGATCCAGGGGAACCCTCAGGGCTGGAGACTCTGAAAGACACACCGCCCCCACGGCT   311
   Chimp1A3 CGGGTGCCCTTCCTTGAGGTCAATGATCCAGGGGAACCCTCAGGGCTGGAGACTCTGAAAGACACACCGCCCCCACGGCT   311
 Gorilla1A3 CGGGTGCCCTTCCTTGAGGTCAATGATCCAGGGGAACCCTCAGGGCTGGAGACTCTGAAAGACACACCGCCCCCACGGCT   311
   Human1A2 CGGGTGCCCTTCCTTGAGTTCAAAGTCCCAGGGATTCCCTCAGGGATGGAGACTCTGAAAAACACACCAGCCCCACGACT   311
   Chimp1A2 CGGGTGCCCTTCCTTGAGTTCAAAGTCCCAGGGATTCCCTCAGGGCTGGAGACTCTGAAAGACACACCGGCCCCACGACT   311
 Gorilla1A2 CGGGTGCCCTTCCTTGAGTTCAAAGTCCCAGGGATTCCCTCAGGGATGGAGACTCTGAAAGACACAGCAGCACCACGACT   311
   Human1A1 CGGGTGCCCTTCCTTGAGTTCAAAGCCCCAGGGATTCCCTCAGGGATGGAGACTCTGAAAGACACACCGGCCCCACGACT   311
   Chimp1A1 CGGGTGCCCTTCCTTGAGTTCAAAGCCCCAGGGATTCCCTCAGGGATGGAGACTCTGAAAGACACACCGGCCCCACGACT   311
 Gorilla1A1 CGGGTGCCCTTCCTTGAGTTCAAAGTCCCAGGGATTCCCTCAGGGCTGGAGACTCTGAAAGACACACCGGCTCCACGGCT   311
  Macaca1A1 CGGGTGCCCTTCCTTGAGTTCAAAGTCCCAGGGATTCCCTCAGGGATGGAGACTCTGAAAGACACACCGGCCCCACGGCT   311
  Rabbit1A1 CGGGTGCCCTTCCTTGAGTTCAAGGCCCCAGGAGCCCCCTCAGGTATGGAGACCCTGAAAGATACACCGTCCCCGCGGCT   311
     Dog1A1 CGGGTACCCTTCCTTGAGTTCAAGGCTCCAGGGATTCCCACAGGTTTGGAGGTTCTAAAAGATACACCAGCCCCACGACT   311
   Mouse1A1 CGGATACCCTTCCTTGAGTTCAGCTGCCCAGGGGTCCCCCAGGTCTTGAAACTCTGAAAGAGACACCAGCCCCACGGAT   320
     Rat1A1 CGGGTACCCTTCCTTGAGTTCAAATGTCCAGGGGTTCCCTCAGGTCTTGAAACTTTGGAAGAGACACCAGCCCCACGGCT   299
     Cow1A1 CGGGTGCCCTTCCTTGAGTTCAGCGCCCCTGGGGTTCCCACAGGTGTTGAGCTTCTGAAAGATACACCAGCCCCACGGCT   311
     Pig1A1 CGGGTGCCCTTCCTTGAGTTCAAAATCCCCAGGTGTCCCACAGGTTTTGAGCTGCTGAAAGATACACCAGCCCCACGGCT   311
Platypus1A1 CGGGTGCCATTTCTGGAGTTCAGCATCCCGGGGATGCCCTCAGGCATGGAGACCTTGAAGGACACTCCCTCCCCACGTCT   311
      ruler .......250.......260.......270.......280.......290.......300.......310.......320

               * *** * **  ****  ** *  * ** **  **    *  **** ** **  *******  ****  * ** ** *
   Human1A4 CATCAAGTCACACCTGCCCCTGGCTCTGCTCCCTCAGACTCTGTTGGATCAGAAGGTCAAGGTGGTCTATGTTGCCCGAA   391
   Human1A3 CATCAAGTCACACCTGCCCCTGGCTCTGCTCCCTCAGACTCTGTTGGATCAGAAGGTCAAGGTGGTCTATGTTGCCCGAA   391
   Chimp1A3 CATCAAGTCACACCTGCCCCTGGCTCTGCTCCCTCAGACTCTGTTGGATCAGAAGGTCAAGGTGGTCTATGTTGCCCGAA   391
 Gorilla1A3 CATCAAGTCACACCTGCCCCTGGCTCTGCTCCCTCAGACTCTGTTGGATCAGAAGGTCAAGGTGGTCTATGTTGCCCGAA   391
   Human1A2 CCTGAAGACACACCTGCCCCTGGCTCTGCTCCCCCAGACTCTGTTGGATCAGAAGGTCAAGGTGGTCTATGTTGCCCGCA   391
   Chimp1A2 CCTGAAGACACACCTGCCCCTGGCTCTGCTCCCCCAGACTCTGTTGGATCAGAAGGTCAAGGTGGTCTATGTTGCCCGCA   391
 Gorilla1A2 CCTGAAGACACACCTGCCCCTGGCTCTGCTCCCCCAGACTCTGTTGGATCAGAAGGTCAAGGTGGTCTATGTTGCCCGCA   391
   Human1A1 CCTGAAGACACACCTGCCCCTGGCTCTGCTCCCCCAGACTCTGTTGGATCAGAAGGTCAAGGTGGTCTATGTTGCCCGCA   391
   Chimp1A1 CCTGAAGACACACCTGCCCCTGGCTCTGCTCCCCCAGACTCTGTTGGATCAGAAGGTCAAGGTGGTCTATGTTGCCCGCA   391
 Gorilla1A1 CATCAAGACACACCTGCCCCTGGCTCTGCTCCCCCAGACTCTGTTGGATCAGAAGGTCAAGGTGGTCTATGTTGCCCGCA   391
  Macaca1A1 CCTGAAGACACACCTGCCCCTGGCCCTGCTGCCCCAGACTCTGTTGGATCAGAAGGTCAAGGTGGTCTATGTTGCCCGCA   391
  Rabbit1A1 CCTCAAGACGCACCTGCCCCTGGCTCTGCTCCCCCAGACTCTCCTGGACCAGAAGGTCAAGGTGGTCTACGTTGCCCGCA   391
     Dog1A1 CATCAAGACACACCTGCCCCTGGCCCTGCTCCCCCAGACCTTGTTGGATCAGAAGGTCAAGGTGGTCTACGTCGCCCGCA   391
   Mouse1A1 CATTAAGACACATCTGCCCTTGTCCTTACTCCCTCAGAGTCTGCTGGATCAAAAGATCAAGGTGATCTACGTTGCCCGAA   400
     Rat1A1 CCTTAAGACACATCTGCCCCTGTCCTTGCTCCCTCAGAGTCTGCTGGATCAGAAGGTCAAGGTGATCTACATTGCCCGAA   379
     Cow1A1 CCTCAAGACACACTTGCCTCTGGCCCTGCTTCCGAAGACCCTGCTGGATCAGAAAGTCAAGGTGATCTACATCGCCCGCA   391
     Pig1A1 CCTCAAGACACACTTGCCCCTGACCCTGCTACCCCAGACTCTGCTGGACCAGAAGGTCAAGGTGGTCTACGTCGCCCGCA   391
Platypus1A1 ACTCAAGACACACCTGCCCTTGGCCTTGCTCCCCAAGGTCTTGCTGGATCAGAAGGTCAAGGTCATCTACATAGCTCGAA   391
      ruler .......330.......340.......350.......360.......370.......380.......390.......400

            *  * ** ** ** *  ** ***** **  * *** *    ****  *      ****  * ** *  ** ***** * *
   Human1A4 ACCCAAAGGACGTGGCGGTCTCCTACTACCATTTCCACCGTATGGAAAAGGCGCACCCTGAGCCTGGGACCTGGGACAGC   471
   Human1A3 ACCCAAAGGACGTGGCGGTCTCCTACTACCATTTCCACCGTATGGAAAAGGCGCACCCTGAGCCTGGGACCTGGGACAGC   471
   Chimp1A3 ACCCAAAGGACGTGGCGGTCTCCTACTACCATTTCCACCGTATGGAAAAGGCGCACCCTGAGCCTGGGACCTGGGACAGC   471
 Gorilla1A3 ACCCAAAGGACGTGGCGGTCTCCTACTACCACTTCCACCGTATGGAAAAGGCGCACCCTGAGCCTGGGACCTGGGACAGC   471
   Human1A2 ACGCAAAGGATGTGGCGGTTTCCTACTACCACTTCTACCACATGGCCAAAGTGTACCCTCACCCTGGGACCTGGGAAAGC   471
   Chimp1A2 ACGCAAAGGATGTGGCGGTTTCCTACTATCACTTCTACCACATGGCCAAAGTGTACCCTCACCCTGGGACCTGGGAAAGC   471
 Gorilla1A2 ACGCAAAGGATGTGGCGGTTTCCTACTACCACTTCTACCACATGGCCAAAGTGTACCCTCACCCTGGGACCTGGGAAAGC   471
   Human1A1 ACGCAAAGGATGTGGCAGTTTCCTACTACCACTTCTACCACATGGCCAAGGTGCACCCTGAGCCTGGGACCTGGGACAGC   471
   Chimp1A1 ACGCAAAGGATGTGGCGGTTTCCTACTACCACTTCTACCACATGGCCAAGGTCCACCCTGAGCCTGGGACCTGGGACAGC   471
 Gorilla1A1 ACGCAAAGGATGTGGCGGTTTCCTACTACCACTTCTACCACATGGACAAGGTGCACCCTGAGCCTGGGACCTGGGACAGC   471
  Macaca1A1 ACGCAAAGGATGTGGCGGTCTCCTACTACCACTTCTACCACATGGCCAAGGTGCACCCCGAGCCTGGGACCTGGGACAGC   471
  Rabbit1A1 ATGCGAAGGATGTGGCCGTCTCCTATTACAATTTCTACCACATGGCCAGGGTGCACCCTGAGCCTGGCACCTGGGACAGC   471
     Dog1A1 ACGCAAAAGATGTAGCTGTCTCCTATTACCACTTCTACCGCATGGCCAAGGTGCACCCTGACCCTGACACCTGGGACAGC   471
   Mouse1A1 ATGCAAAGGATGTGGTTGTCTCCTATTATAACTTCTACAAAATGGCCAAGCTGCACCCTGACCCAGGCACCTGGGAAAGC   480
     Rat1A1 ATGCAAAGGATGTGGTTGTCTCCTATTATAACTTCTACAACATGGCCAAGCTGCACCCTGATCCAGGCACCTGGGACAGC   459
     Cow1A1 ACGCCAAGGATGTGGCCGTCTCCTATTACCACTTCTATCGCATGGCCAAGGTGCACCCTGACCCTGGCACCTGGGACAGC   471
     Pig1A1 ACGCAAAGGACGTGGCCGTCTCCTATTACCACTTCTACCGTATGGCCAAAGTGTACCCTAACCCTGGCACGTGGGACAGC   471
Platypus1A1 ACGCCAAGGACGTGGCCGTGTCCTACTACCATTTCTATCGAATGGCTAAAGTGCACCCCGATCCGGGCACTTGGGAGACC   471
      ruler .......410.......420.......430.......440.......450.......460.......470.......480
```

```
            **  * **    ** *  *  **  **** *  ** ** ** ***** ***** **    ** ******** *** *
   Human1A4 TTCCTGGAAAAGTTCATGGCTGGAGAAGTGTCCTACGGGTCCTGGTACCAGCACGTGCAGGAGTGGTGGGAGCTGAGCCG   551
   Human1A3 TTCCTGGAAAAGTTCATGGCTGGAGAAGTGTCCTACGGGTCCTGGTACCAGCACGTGCAGGAGTGGTGGGAGCTGAGCCG   551
   Chimp1A3 TTCCTGGAAAAGTTCATGGCTGGAGAAGTGTCCTACGGGTCCTGGTACCAGCACGTGCAGGAGTGGTGGGAGCTGAGCCG   551
 Gorilla1A3 TTCCTGGAAAAGTTCATGGCTGGAGAAGTGTCCTACGGGTCCTGGTACCAGCACGTGCAGGAGTGGTGGGAGCTGAGCCG   551
   Human1A2 TTCCTGGAGAAGTTCATGGCTGGAGAAGTGTCCTATGGGTCCTGGTACCAGCACGTGCAAGAGTGGTGGGAGCTGAGCCG   551
   Chimp1A2 TTCCTGGAGAAGTTCATGGCTGGAGAAGTGTCCTATGGGTCCTGGTACCAGCACGTGCAGGAGTGGTGGGAGCTGAGCCG   551
 Gorilla1A2 TTCCTGGAGAAGTTCATGGCTGGAGAAGTGTCCTATGGGTCCTGGTACCAGCACGTGCAGGAGTGGTGGGAACTGAGCCG   551
   Human1A1 TTCCTGGAGAAGTTCATGGTCGGAGAAGTGTCCTACGGATCCTGGTACCAGCACGTGCAGGAGTGGTGGGAGCTGAGCCG   551
   Chimp1A1 TTCCTGGAGAAGTTCATGGTCGGAGAAGTGTCCTATGGATCCTGGTACCAGCACGTGCAGGAGTGGTGGGAGCTGAGCCG   551
 Gorilla1A1 TTCCTGGAGAAGTTCATGGTCGGAGAAGTGTCCTACGGATCCTGGTACCAGCACGTGCAGGAGTGGTGGGAGCTGAGCCG   551
  Macaca1A1 TTCCTGGAGAAGTTCATGGCCGGAGAAGTGTCCTATGGGTCCTGGTACCAGCACGTGCAGGAGTGGTGGGAGCTGAGCCA   551
  Rabbit1A1 TTCCTGGAGAAGTTCATGGCCGGGAAAGTGTCCTACGGCTCCTGGTACCAGCACGTGCGGGAGTGGTGGGAGCTGAGCCG   551
     Dog1A1 TTCCTGGAGAAGTTCATGGCTGGGGAAGTGTCCTATGGGTCCTGGTATCAGCATGTGCAGGAATGGTGGGAGCTGAGTCA   551
   Mouse1A1 TTCTTGGAGAACTTCATGGATGGGAAAGTGTCCTATGGGTCGTGGTACCAGCACGTGAAGGAGTGGTGGGAGCTGAGACG   560
     Rat1A1 TTCTTGGAGAACTTCATGGATGGGGAAGTGTCCTATGGGTCGTGGTACCAGCACGTGAAGGAGTGGTGGGAGCTGAGACA   539
     Cow1A1 TTCCTGGAGAAGTTCATGGCCGGGGAAGTGTGCTACGGGTCCTGGTACCAGCACGTGCAGGAGTGGTGGGAGCTGAGTCA   551
     Pig1A1 TTCCTGGAAGACTTCATGGCCGGAGAAGTGTCCTACGGGTCCTGGTACCAGCACGTGCAGGAGTGGTGGGAGCTGCGTCA   551
Platypus1A1 TTTCTAGAGGCATTTAAAGCCGGGCAAGTTTGTTACGGGTCCTGGTACCAGCACGTCCAAGACTGGTGGGAACTGAGGAA   551
      ruler .......490.......500.......510.......520.......530.......540.......550.......560

                ** ** ** ***** *******  ** ***** ** *** * ** **  *******    *****  *  ****
   Human1A4 CACCCACCCTGTTCTCTACCTCTTCTATGAAGACATGAAGGAGAACCCCAAAAGGGAGATTCAAAAGATCCTGGAGTTTG   631
   Human1A3 CACCCACCCTGTTCTCTACCTCTTCTATGAAGACATGAAGGAGAACCCCAAAAGGGAGATTCAAAAGATCCTGGAGTTTG   631
   Chimp1A3 CACCCACCCTGTTCTCTACCTCTTCTATGAAGACATGAAGGAGAACCCCAAAAGGGAGATTCAAAAGATCCTGGAGTTTG   631
 Gorilla1A3 CACCCACCCTGTTCTCTACCTCTTCTATGAAGACATGAAGGAGAACCCCAAAAGGGAGATTCAAAAGATCCTGGAGTTTG   631
   Human1A2 CACCCACCCTGTTCTCTACCTCTTCTATGAAGACATGAAGGAGAACCCCAAAAGGGAGATTCAAAAGATCCTGGAGTTTG   631
   Chimp1A2 CACCCACCCTGTTCTCTACCTCTTCTATGAAGACATGAAGGAGAACCCCAAAAGGGAGATTCAAAAGATCCTGGAGTTTG   631
 Gorilla1A2 CACCCACCCTGTTCTCTACCTCTTCTATGAAGACATGAAGGAGAACCCCAAAAGGGAGATCCAAAAGATCCTGGAGTTTG   631
   Human1A1 CACCCACCCTGTTCTCTACCTCTTCTATGAAGACATGAAGGAGAACCCGAAAAGGGAGATTCAAAAGATCCTGGAGTTTG   631
   Chimp1A1 CACCCACCCTGTTCTCTACCTCTTCTATGAAGACATGAAGGAGAACCCCAAAAGGGAGATTCAAAAGATCCTGGAGTTTG   631
 Gorilla1A1 CACCCACCCTGTTCTCTACCTCTTCTATGAAGACATGAAGGAGAACCCCAAAAGGGAGATTCAAAAGATCCTGGAGTTTG   631
  Macaca1A1 CACCCACCCTGTTCTCTACCTCTTCTATGAAGACATGAAGGAGAACCCCAAAAGGGAGATTTGGAAGATCCTGGAGTTCG   631
  Rabbit1A1 CACCCACCCCGTCCTCTACCTCTTCTTTGAGGACATGAAGGAGAACCCCAAGAGGGAGATCAAGAAGATCCTGGAGTTCC   631
     Dog1A1 CACTCACCCTGTTCTCTACCTCTTCTATGAGGACATGAAAGAGAACCCCAAAAGGGAGATTCAGAAGATCCTGAAGTTTG   631
   Mouse1A1 CACTCACCCTGTTCTCTATCTCTTCTATGAAGACATGAAGGAGAATCCCAAAAGGGAGATCAAGAAGATTCTAGAGTTTC   640
     Rat1A1 CACTCACCCTGTTCTCTATCTCTTCTATGAAGACATAAAGGAGAACCCCAAAAGGGAGATCAAGAAGATTCTAGAGTTTT   619
     Cow1A1 CACCCACCCTGTTCTCTACCTCTTCTACGAGGACATAAAGGAGGACCCCAAAAGGGAGATTCAGAAGATCCTGGAGTTTA   631
     Pig1A1 CACCCACCCTGTCCTCTATCTCTTCTACGAGGACATGAAGGAGAACCCCAAGAGGGAGATTCAGAAGATCCTGGAGTTTG   631
Platypus1A1 GCAGCAACCGGTCCTCTACCTCTTCTTCGAAGACATAAAGGAGGATCCTAAGCGGGAGATCAGAAAGATCATGGAGTTCA   631
      ruler .......570.......580.......590.......600.......610.......620.......630.......640

            * *  *      *  * *******   * **    ** *   *    ** ** *** *  * *****  * **    ***
   Human1A4 TGGGGCGCTCCCTGCCAGAGGAGACCATGGACTTCATGGTTCAGCACACGTCGTTCAAGGAGATGAAGAAGAACCCTATG   711
   Human1A3 TGGGGCGCTCCCTGCCAGAGGAGACCATGGACTTCATGGTTCAGCACACGTCGTTCAAGGAGATGAAGAAGAACCCTATG   711
   Chimp1A3 TGGGGCACTCCCTGCCAGAGGAGACCATGGACTTCATGGTTCAGCACACGTCGTTCAAGGAGATGAAGAAGAACCCTATG   711
 Gorilla1A3 TGGGGCGCTCCCTGCCAGAGGAGACCGTGGACTTCATGGTTCAGCACACGTCGTTCAAGGAGATGAAGAAGAACCCTATG   711
   Human1A2 TGGGGCGCTCCCTGCCAGAGGAGACTGTGGACCTCATGGTTGAGCACACGTCGTTCAAGGAGATGAAGAAGAACCCTATG   711
   Chimp1A2 TGGGGCGCTCCCTGCCAGAGGAGACCGTGGACCTCATGGTTGAGCACACGTCGTTCAAGGAGATGAAGAAGAACCCTATG   711
 Gorilla1A2 TGGGGCGCTCCCTGCCAGAGGAGACTGTGGACCTCATGGTTGAGCACACGTCGTTCAAGGAGATGAAGAAGAACCCTATG   711
   Human1A1 TGGGGCGCTCCCTGCCAGAGGAGACCGTGGACTTCATGGTTCAGCACACGTCGTTCAAGGAGATGAAGAAGAACCCTATG   711
   Chimp1A1 TGGGGCGCTCCCTGCCAGAGGAGACCGTGGACTTCATGGTTCAGCACACGTCGTTCAAGGAGATGAAGAAGAACCCTATG   711
 Gorilla1A1 TGGGGCGCTTCCTGCCAGAGGAGACCGTGGACCTCATGGTTCAGCACACGTCGTTCAAGGAGATGAAGAAGAACCCTATG   711
  Macaca1A1 TGGGGCGCTCCCTGCCAGAGGAGACCGTAGACCTCATGGTTCAACACACGTCATTCAAGGAGATGAAGAAGAACCCTATG   711
  Rabbit1A1 TGGGGCGCTCCCTGCCGGAGGAGACGGTGGACCGCATCGCCCACTGCACGTCTTTCAAGGAGATGAAGAAGAACCCCATG   711
     Dog1A1 TGGGGCGCTCCCTGCCAGAGGAGACTGTGGATCTCATTGTCCAGCACACGTCTTTCAAGGAGATGAAGAACAACTCCATG   711
   Mouse1A1 TGGGGCGCTCTCTACCTGAGGAGACTGTGGATTTAATTGTTCACCACACATCCTTCAAGAAAATGAAGGAGAACCCCATG   720
     Rat1A1 TGGGGCGCTCTCTACCCGAGGAGACTGTGGATTCCATTGTTCACCACACATCTTTCAAGAAAATGAAAGAGAACTGCATG   699
     Cow1A1 TAGGGCGCTCCCTGCCTGAGGAGACTGTGGATCACATTGTCCAGCGCACATCTTTCAAAGAGATGAAGAAGAACCCCATG   711
     Pig1A1 TGGGGCGCTCGCTGCCAGAGGAGACGGTGGAGGACATCGTCCAGCACACGTCCTTCCAGGAGATGAAGAACAACGCCATG   711
Platypus1A1 TAGACCAGCCTGTCTCGGAGGAGATATTGGATCGGATTGTCCAGGAGACATCCTTCAAGAAGATGAAGCAGAATCCCATG   711
      ruler .......650.......660.......670.......680.......690.......700.......710.......720
```

```
             * ******    *  *           *  ********      *   **** *** * * ***       ** ** **
   Human1A4 ACCAACTACACCACCGTCCCCCAGGAGCTCATGGACCACAGCATCTCCCCCTTCATGAGGAAAGGCATGGCTGGGGACTG   791
   Human1A3 ACCAACTACACCACCGTCCCCCAGGAGCTCATGGACCACAGCATCTCCCCCTTCATGAGGAAAGGCATGGCTGGGGACTG   791
   Chimp1A3 ACCAACTACACCACCGTCCCCCAGGAGCTCATGGACCACAGCACCTCCCCCTTCATGAGGAAAGGCATGGCTGGGGACTG   791
 Gorilla1A3 ACCAACTACACCACCGTCCCCCAGGATCTTATGGACCACAGCATCTCCCCCTTCATGAGGAAAGGCATGGCTGGGGACTG   791
   Human1A2 ACCAACTACACCACCGTCCGCCGGGAGTTCATGGACCACAGCATCTCCCCCTTCATGAGGAAAGGCATGGCTGGGGACTG   791
   Chimp1A2 ACCAACTACACCACCGTCCACCGGGAGTTCATGGACCACAGCATCTCCCCCTTCATGAGGAAAGGCATGGCTGGGGACTG   791
 Gorilla1A2 ACCAACTACACCACCGTCCCCAGGGAGTTCATGGACCACAGCATCTCCCCCTTCATGAGGAAAGGCATGGCTGGGGACTG   791
   Human1A1 ACCAACTACACCACCGTCCCCCAGGAGTTCATGGACCACAGCATCTCCCCCTTCATGAGGAAAGGCATGGCTGGGGACTG   791
   Chimp1A1 ACCAACTACACCACCGTCCCCCAGGAGTTCATGGACCACAGCATCTCCCCCTTCATGAGGAAAGGCATGGCTGGGGACTG   791
 Gorilla1A1 ACCAACTACACCACCGTCCCCTGGGAGTTCATGGACCACAGCATCTCCCCCTTCATGAGGAAAGGCATGGCTGGGGACTG   791
  Macaca1A1 GCCAACTACACCACCATCCCCCAGGAGCTCATGGACCACAGCATCTCCCCCTTCATGAGGAAAGGAATGACTGGGGACTG   791
  Rabbit1A1 ACCAACTACAGCACCATCCCCGAAAACATCATGGACCACAACGTCTCCCCCTTCATGCGCAGAGGCGTGGCAGGGGACTG   791
     Dog1A1 GCTAACTACACCACCTTATCTCCTGACATCATGGACCACAGCATTTCTGCCTTCATGAGGAAAGGCATCTCGGGGGACTG   791
   Mouse1A1 GCTAACTACACAACCATCCCAACTGAAGTTATGGACCACACTATTTATCCCTTCATGAGGAAAGGTACCATTGGGGACTG   800
     Rat1A1 ACTAACTACACAACCATCCCCACTGAGATTATGGACCACAATGTTTCTCCCTTCATGAGGAAAGGTACTACTGGGGACTG   779
     Cow1A1 ACCAACTACAGCACCATACCCACTGCAGTCATGGACCACAGCATCTCTGCCTTCATGAGGAAAGGCATCACTGGTGATTG   791
     Pig1A1 ACCAACTACCGCACCCTCCCCTCCGACCTCCTGGACCACAGCATCTCAGCCTTCATGAGGAAAGGCATCACTGGAGATTG   791
Platypus1A1 ACCAACTACAGCTCTGTCCCTTCTCATATCATGGACCACGAGGTCTCCCCCTTTATGAGGAAAGGCACCCCCGGGGACTG   791
      ruler .......730.......740.......750.......760.......770.......780.......790.......800

            ***      ****** ** ** ***   **    ** ** *   **** *   **   ***   *
   Human1A4 GAAGACCACCTTCACCGTGGCGCAGAATGAGCGCTTCGATGCGGACTATGCGGAGAAGATGGCAGGCTGCAGCCTCAGCT   871
   Human1A3 GAAGACCACCTTCACCGTGGCGCAGAATGAGCGCTTCGATGCGGACTATGCGGAGAAGATGGCAGGCTGCAGCCTCAGCT   871
   Chimp1A3 GAAGACCACCTTCACCGTGGCGCAGAATGAGCGCTTCGATGCGGACTATGCGGAGAAGATGGCAGGCTGCAGCCTCAGCT   871
 Gorilla1A3 GAAGACCACCTTCACCGTGGCGCAGAATGAGCGCTTCGATGCGGACTATGCGGAGAAGATGGCAGGCTGCAGTCTCAGCT   871
   Human1A2 GAAGACCACCTTCACCGTGGCGCAGAATGAGCGCTTCGATGCGGACTATGCGGAGAAGATGGCAGGCTGCAGCCTCAGCT   871
   Chimp1A2 GAAGACCACCTTCACCGTGGCGCAGAATGAGCGCTTCGATGCGGACTATGCGGAGAAGATGGCAGGCTGCAGCCTCAGCT   871
 Gorilla1A2 GAAGACCACCTTCACCGTGGCGCAGAATGAGCGCTTCGATGCGGACTATGTGGAGAAGATGGCAGGCTGCAGCCTCAGCT   871
   Human1A1 GAAGACCACCTTCACCGTGGCGCAGAATGAGCGCTTCGATGCGGACTATGCGAAGAAGATGGCAGGCTGCAGCCTCACGT   871
   Chimp1A1 GAAGACCACCTTCACCGTGGCGCAGAATGAGCGCTTCGATGCGGACTATGCGGAGAAGATGGCAGGCTGCAGCCTCAGCT   871
 Gorilla1A1 GAAGACCACCTTCACCGTGGCGCAGAATGAGCGCTTCGATGCGGACTATGCGGAGAAGATGGCAGGCTGCAGCCTCAGCT   871
  Macaca1A1 GAAAACCACCTTCACCGTGGCGCAGAATGAGCACTTCGATGTGGACTATGCGGAGAAGATGGCAGGCTGCAGCCTCAGCT   871
  Rabbit1A1 GAAGACCACCTTCACCGTGGCCCAGCATGAGTACTTTGAGGCCGACTACGCCGAGAAGATGGCGGGCTGCGAGCTCACCT   871
     Dog1A1 GAAGACCACCTTCACTGTGGCCCAGAATGAGCGCTTTGATGCCGACTATGCCAAGAAGATGGAGGGTTGTGGCCTCAGCT   871
   Mouse1A1 GAAAAATACCTTCACTGTAGCCCAGAGTGAGCACTTTGATGCCCACTATGCCAAGCTAATGACAGGTTGTGACTTCACGT   880
     Rat1A1 GAAAAATACCTTCACTGTAGCCCAGAATGAGCGCTTTGATGCCCACTATGCTAAGACAATGACAGATTGTGACTTCAAGT   859
     Cow1A1 GAAATCCACCTTCACTGTGGCCCAGAATGAGCTCTTTGAAGCCCACTATGCTAAGAAGATGCGGGCTGCAACTCCGCT--   869
     Pig1A1 GAAAAGCACCTTCACAGTGGCCCAGAACGAGCGCTTTGAGGCCGACTATGCTGAGAAGATGGCAGGCTGTAACCTCCGCT   871
Platypus1A1 GAAGAACCACTTCACGGTGGCCCAGAATGAACTGTTCGATGTCGACTACGCAGAGAAAATGGCGGGCTCGGAGCTGCACT   871
      ruler .......810.......820.......830.......840.......850.......860.......870.......880

              **     *  *
   Human1A4 TCCGCTCTGAGCTG   885
   Human1A3 TCCGCTCTGAGCTG   885
   Chimp1A3 TCCGCTCTGAGCTG   885
 Gorilla1A3 TCCGCTCTGAGCTG   885
   Human1A2 TCCGCTCTGAGCTG   885
   Chimp1A2 TCCGCTCTGAGCTG   885
 Gorilla1A2 TCCGCTCTGAGCTG   885
   Human1A1 TCCGCTCTGAGCTG   885
   Chimp1A1 TCCGCTCTGAGCTG   885
 Gorilla1A1 TCCGCTCTGAGCTG   885
  Macaca1A1 TCCGCTCTGAGCTG   885
  Rabbit1A1 TCCGTTCAGAGCTG   885
     Dog1A1 TCCGCACACAGCTG   885
   Mouse1A1 TCCGCTGTCAAATA   894
     Rat1A1 TTCGTTGTGAACTA   873
     Cow1A1 -TCGCTGGGAGCTG   882
     Pig1A1 TCCGCTCTGAGCTG   885
Platypus1A1 TCCGCACCGAGCTG   885
      ruler .......890....
```
