# Supplementary material for: Phylogenomic approaches to common problems encountered in the analysis of low copy repeats: The sulfotransferase 1A gene family example
Source: BMC Evol Biol. 2005 Mar 7;5:22. doi: 10.1186/1471-2148-5-22 (PMC555591; doi:10.1186/1471-2148-5-22)
Supplement: Additional File 2 — Pairwise n and c values for SULT1A genes. Pairwise n and c values between SULT1A genes. The names of the sequences are the row-headers and the column-headers. Lower triangular matrix contains n values, and upper triangular matrix contains c values. [file 1471-2148-5-22-S2.pdf]

Supplementary Table 1

Pairwise *n* and *c* Values for SULT 1A Genes

	Human 1A4	Human 1A3	Chimp 1A3	Gorilla 1A3	Human 1A2	Chimp 1A2	Gorilla 1A2	Human 1A1	Chimp 1A2	Gorilla 1A1	Macaca	Rabbit	Dog	Mouse	Rat	Pig	Cow	Platypus
Human 1A4		118	118	116	96	98	98	106	105	103	99	81	79	63	68	80	76	63
Human 1A3	119		117	115	97	99	99	107	106	104	99	81	78	63	68	79	76	63
Chimp 1A3	118	118		115	96	97	97	105	104	102	98	81	78	63	67	80	75	62
Gorilla 1A3	117	117	116		95	97	97	105	104	103	98	80	80	63	67	80	76	61
Human 1A2	104	104	103	102		110	112	106	107	105	100	81	79	67	70	79	74	61
Chimp 1A2	106	106	105	104	114		112	106	109	105	100	82	78	67	71	79	74	60
Gorilla 1A2	105	105	104	103	115	115		108	109	107	102	80	80	66	70	79	75	63
Human 1A1	110	110	109	108	110	110	111		115	113	106	84	85	68	74	80	82	63
Chimp 1A1	111	111	110	109	111	111	112	117		112	107	85	84	67	73	80	80	63
Gorilla 1A1	109	109	108	107	111	111	112	115	116		104	83	84	67	73	79	81	62
Macaca	108	108	107	106	107	107	108	112	113	112		82	85	68	74	78	80	61
Rabbit	95	95	96	94	94	96	95	97	98	98	98		72	65	65	80	72	56
Dog	100	100	99	100	100	100	101	105	104	104	103	91		68	71	79	81	54
Mouse	85	85	85	84	86	87	86	87	86	86	87	86	89		99	60	64	44
Rat	89	89	88	88	89	90	89	92	91	91	92	88	92	107		64	68	47
Cow	98	98	99	98	100	100	101	101	102	102	101	93	101	81	86		80	54
Pig	95	95	94	94	94	94	95	99	98	98	98	89	99	82	87	97		57
Platypus	85	85	84	84	86	86	87	87	88	88	88	82	88	79	80	86	91	
